# Development of a SimpleProbe real-Time PCR Assay for rapid detection and identification of the US novel urethrotropic clade of *Neisseria meningitidis* ST-11 (US_NmUC)

**DOI:** 10.1371/journal.pone.0228467

**Published:** 2020-02-10

**Authors:** Evelyn Toh, James A. Williams, Brahim Qadadri, Aaron Ermel, David E. Nelson

**Affiliations:** 1 Department of Microbiology and Immunology, Indiana University School of Medicine, Indianapolis, Indiana, United States of America; 2 Department of Medicine, Division of Infectious Diseases, Indiana University School of Medicine, Indianapolis, Indiana, United States of America; Public Health England, UNITED KINGDOM

## Abstract

Urethritis, or inflammation of the urethra, is one of the most common reasons men seek clinical care. Sexually transmitted pathogens including *Neisseria gonorrhoeae* are responsible for over half of the symptomatic urethritis cases in U.S. men. Recently, clinics in Indianapolis, Columbus, Atlanta, and other U.S. cities began to note increasing numbers of men presenting with urethritis and Gram-negative intracellular diplococci in their urethral smears who test negative for *N*. *gonorrhoeae*. Many of these discordant cases, which have periodically reached highs of more than 25% of presumed gonococcal cases in some sexually transmitted infection clinics in the U.S. Midwest, are infected with strains in a novel urethrotropic clade of *Neisseria meningitidis* ST-11 (US_NmUC). However, no cultivation-independent tests are available for the US_NmUC strains, and prior studies relied on microbial culture and genome sequencing to identify them. Here, we describe a PCR test that can identify the US_NmUC strains and distinguish them from commensal and invasive *N*. *meningitidis* strains as well as *N*. *gonorrhoeae*. Our SimpleProbe®-based real-time PCR assay targets a conserved nucleotide substitution in a horizontally acquired region of US_NmUC strain genomes. We applied the assay to 241 urine specimens whose microbial compositions had previously been determined by deep shotgun metagenomic sequencing. The assay detected the single US_NmUC positive case in this cohort, with no false positives. Overall, our simple and readily adaptable assay could facilitate investigation of the pathogenesis and epidemiology of the US_NmUC clade.

## Introduction

Urethritis is defined by the presence of polymorphonuclear cells in male urethral smear or urine specimens, and is often, but not always, associated with urethral and urinary symptoms [1–4]. Millions of cases of urethritis occur in men in the U.S. each year, and urethritis-related symptoms are one of the most common reasons men seek primary care [5]. Infections with a number of known sexually transmitted bacterial, viral, and protozoal pathogens can elicit urethritis, but many cases are idiopathic [1, 6, 7].

Many specialized sexually transmitted infection (STI) clinics use point-of-care urethral Gram stain smears to categorize urethritis into gonococcal urethritis (GU) and nongonococcal urethritis (NGU) cases because the optimal empiric antibiotic regimens for GU and NGU differ [1, 8]. Presence of Gram-negative intracellular diplococci in these smears is highly sensitive and specific for *Neisseria gonorrhoeae* (NG). Men with urethritis whose Gram stain smears contain Gram-negative intracellular diplococci are diagnosed with GU, whereas men whose smears who do not are diagnosed with NGU. When a point-of-care test is unavailable, a negative nucleic acid amplification test for NG can indicate a diagnosis of NGU. *Chlamydia trachomatis*, *Mycoplasma genitalium*, and *Trichomonas vaginalis* are common causes of NGU [9–12]. Sensitive and specific nucleic acid amplification tests are available for some pathogens that cause NGU. However, syndromic management of urethritis based on Gram stain results is the norm, and identification of NGU-associated pathogens, when attempted, is usually retrospective [2].

*Neisseria meningitidis* (NM) is best known for its ability to invade and cause meningitis and septicemia, although these invasive infections are rare and asymptomatic nasopharyngeal NM colonization is more common [13, 14]. NM has previously been associated with single cases and small clusters of urethritis cases [15–22], but none of these earlier outbreaks were sustained.

Multiple STI clinics in the U.S. Midwest began to note an increased frequency of Gram-negative intracellular diplococci-positive, NG nucleic acid amplification test-negative, male urethritis cases starting in 2015 [23–25]. Urethral specimens from some of these men grew NM [23–25], and whole genome sequencing revealed that the isolates were members of a new, and nearly clonal, sub-group of strains in the ST-11 clonal complex, sub-lineage 11.2 [26]. These urethrotropic NM ST-11 (US_NmUC) isolates are a new paradigm because they have an insertion in the capsule biosynthesis locus and can grow anaerobically due to horizontal acquisition of a region of the *norB* (PubMLST allele NEIS1548*)-aniA* (PubMLST allele NEIS1549) locus from NG [25–27]. The emergence of US_NmUC is alarming because this clade only diverged from invasive NM ST-11 strains recently, and its pathogenic potential and epidemiology have not been defined [28]. US_NmUC sub-lineages have acquired DNA from NG on multiple occasions since their divergence from an invasive NM ST-11 parent, so there is also concern that these strains could acquire additional genes from NG that confer resistance to antibiotics and host immune defenses [26, 28, 29].

Nucleic acid amplification tests have mostly supplanted cultivation for diagnosis of NG, *C*. *trachomatis*, *T*. *vaginalis*, and *M*. *genitalium* because culture of these microorganisms is costly, requires controlled transport conditions, and FDA-approved tests that offer increased sensitivity compared to culture of these organisms are now widely available. NM is not routinely cultured from urogenital sites, and since the sensitivity of NM culture from routine oropharyngeal specimens using optimized transport conditions can be as low as 72% [30], the sensitivity of urogenital NM culture may also be low. However, no cultivation-independent tests that can detect and differentiate US_NmUC from other NM strains have been reported. In prior studies, US_NmUC was usually cultured from urogenital specimens in settings where there was a high degree of clinical suspicion due to discordant Gram stain smear and NG test results, and then was identified using multi-locus sequence typing and or whole genome sequencing. Culture and whole genome sequencing are not available in primary STI care settings where most urethritis cases present, so US_NmUC prevalence has been estimated from a handful of studies of symptomatic male STI clinic attendees and retrospective analyses of banked isolates.

Here, we describe a rapid real-time test for the US_NmUC clade strains using a SimpleProbe® assay [31]. The assay probe targets a single nucleotide polymorphism in *norB* that is present in US_NmUC clade strains, but absent in invasive NM strains, NG, and other commensal *Neisseria* species that colonize the urogenital tract. Assay sensitivity and specificity were evaluated using urine and urogenital swab matrices spiked with urogenital pathogens, representative US_NmUC clade isolates, and other pathogenic and commensal urogenital microorganisms. The assay also identified the only US_NmUC positive specimen in a collection of 241 male urine specimens whose microbiomes had been characterized by deep shotgun metagenomic sequencing. The US_NmUC Simpleprobe® assay could be easily adapted for other types of clinical specimens, is compatible with platforms used in contemporary diagnostic laboratories, and can be rapidly implemented for US_NmUC surveillance.

## Materials and methods

### Bacterial strains, plasmids and DNA templates

Unpublished NG and NM clinical isolates from Indianapolis area clinics were used to represent local strains; other strains were obtained from American Type Culture Collection (ATCC) and other sources. NM FAM18 (ATCC 700532D-5), NM1 and NM2 [25]; NM3 and NM4 (Indianapolis clinical isolates); ATL1 and ATL2 [26]; CNM14, CNM17, CNM32, CNM33, CNM37, and CNM45 [24]; NG1 and NG2 (Indianapolis clinical isolates); *Neisseria perflava* (ATCC-14799); *Neisseria subflava* (ATCC 49275); *C*. *trachomatis* [32]; *M*. *genitalium* [32]; *T*. *vaginalis* [32]; and *Ureaplasma urealyticum*, (Ken Waites, University of Alabama at Birmingham). HSV 1 and 2 control DNA was purchased from Acrometrix (Thermo Fisher Scientific Life Sciences, Waltham, MA) and the HPV 16 plasmid was a gift from the Indiana University Infectious Disease laboratory.

### Collection and characterization of clinical specimens

Males ≥18 years of age who presented to the Marion County Public Department of Health, Bellflower STI Clinic (BFC) in Indianapolis, Indiana with acute urethral symptoms or as healthy controls without symptoms, and provided written consent to participate, were enrolled in the idiopathic urethritis men’s project (IUMP) [12, 33]. A Gram stain smear was prepared from a urethral swab from each participant to evaluate the presence of Gram-negative intracellular diplococci and determine the number of polymorphonuclear leukocytes per high-power field. We collected first-catch urine from the participants for US_NmUC testing. The men were diagnosed with NGU if they had ≥2 polymorphonuclear leukocytes per high-power field by urethral Gram stain smear and or urethral discharge on physical exam (N = 127). Asymptomatic men without urethral discharge and <2 polymorphonuclear leukocytes per high-power field by urethral Gram stain smear were identified as healthy controls (N = 114). Total DNA from the urethral swabs was extracted and dual-indexed sequencing libraries were constructed using the NexteraXT DNA Library preparation kit (Illumina, San Diego, CA). Sequencing libraries were pooled 12 per lane, and sequenced on an Illumina HiSeq 4000, generating 150 base paired-end sequences. Microorganism sequences were annotated using MetaPhlan2 [34]. NM sequences were detected in the urethral specimen from one man with idiopathic NGU, and the presence of US_NmUC in his corresponding urine was confirmed by PCR amplification and sequencing of the *norB-aniA* locus. The Indiana University/Purdue University-Indianapolis Institutional Review Board and the Marion County Public Department of Health approved this study.

### SimpleProbe® US_NmUC real-time PCR assay

A primer set was designed with Roche Applied Science Light Cycler Probe design software 2.0 to amplify a 117 bp segment of the 2,256 bp *norB* gene that contains the target SNP in position 431 in the US_NmUC reference isolate NM1 [25]. A SimpleProbe® probe PNM1 (Fluorescein-SPC-CGTCATCAGCGATACGCG-Phosphate) was synthesized by FLUORESENTRIC (Park City, UT). The PCR reaction mixtures contained LightCycler 480 Genotyping Master Mix (2x) (Roche Diagnostics, Indianapolis, IN), 0.5 μm of the forward primer F-NM1 (5’-GCTTGGCCGATGAATACC-3’), 1.5 μM of the reverse primer R-NM1 (5’-CGTAAACGCCGTGATAGT-3’), 0.2 μM probe (P-NM1), 5 or 2 μl DNA template, and 3 mM MgCl_2_ in a total reaction volume of 20 μl. Amplifications were initially performed on a Roche LightCycler 2.0 instrument and were subsequently scaled-up on a Roche z480 (z480) real-time instrument (Roche Diagnostics, Indianapolis, IN). Amplification conditions for the LightCycler 2.0 were: 95°C for 10 min, 45 cycles at 95°C for 10 sec (ramp rate of 20°C/s), 55°C for 20 sec in single acquisition mode (ramp rate of 10°C/s), 72°C for 20 sec (ramp rate of 20°C/s). Melting curve analysis commenced by raising the temperature to 95°C for 0 sec (ramp rate of 20°C/s), followed by annealing at 40°C for 2 min (ramp rate of 1.5°C/s). The temperature was increased stepwise to 95°C with continuous signal acquisition (ramp rate of 0.1°C/s). Cool down was performed at 40°C for 30 sec (ramp rate 20°C/s). Amplification conditions on the z480 were: 95°C for 10 min (ramp rate 4.4°C/s), 45 cycles at 95°C for 10 sec (ramp rate of 4.4°C/s), 55°C for 20 sec in single acquisition mode (ramp rate 2.2°C/s), 72°C for 20 sec (ramp rate 4.4°C/s). After amplification, melting curve analysis commenced by raising the temperature to 95°C for 1 min (ramp rate 4.4°C/s), followed by annealing at 40°C for 2 min (ramp rate 2.2°C/s). In the final annealing step, the temperature was raised to 90°C (ramp rate 0.06°C/s) with continuous signal acquisition. Cool down was performed at 40°C for 30 sec (ramp rate 2.2°C/s).

### Determination of analytical sensitivity

To construct the control plasmids, 400 bp amplicons of *norB* encompassing the region containing the target SNP were PCR amplified from NM1 or NG genomic DNA. The amplicons were cloned into the pCR2.1-TOPO vector (Thermo Fisher Scientific Life Sciences, Waltham, MA, USA) to create pNG-*norB* and pNM1-*norB*. The sequences of the *norB* inserts in the final plasmids were confirmed by sequencing. The limit of detection (LOD) of the SimpleProbe® US_NmUC real-time assay was determined using spiked mock urine and vaginal swab matrices that tested negative for NG, *C*. *trachomatis*, *T*. *vaginalis*, *M*. *genitalium*, and *U*. *urealyticum*. Serial dilutions of plasmids pNG-*norB* and pNM1-*norB* were added directly to mock urine and vaginal swab solutions, with the final plasmid concentrations ranging from 25 to 1000 copies/ml in the spiked urine matrices, and 250–4000 copies/ml in the spiked vaginal swab matrices. The contrived samples were processed, and DNA was extracted on the Roche Cobas 4800 automated extraction platform.

### Taqman assay for Neisserial *metA*

A TaqMan assay for the NM *metA* gene was adapted from a prior study [35]. The assay was scaled for use with the User Defined Workflow Software version 2.0 using the z480 platform.

## Results and discussion

### Assay design and validation using control strains

The US_NmUC clade recently diverged from an invasive ST-11 parent, and lineages of this clade have subsequently recombined with other NM strains, so few genomic features can reliably differentiate urethrotopic from non-urethrotropic NM ST-11 isolates. However, at least two key genetic changes drove the emergence of the US_NmUC clade: disruption of the capsule locus by an insertion sequence (IS) element and acquisition of a region of the *norB-aniA* locus via a horizontal gene transfer event with NG [25, 26, 28]. We rejected targeting the IS element in the capsule locus because this region has the potential to recombine with other IS elements present in US_NmUC isolates [26], although recombination at this specific site has not yet been documented. We identified orthologs of gonococcal *norB* in all 204 of the US_NmUC clade strains for which complete genomes were available (26). The *norB* sequences were identical in 194 of these 204 isolates. Alignment of the *norB-ani* locus of NM1, NG, NM FAM18, and other commensal *Neisseria* spp. revealed two nonsynonymous SNPs in *norB*, G431A and C1782T, that were unique to NM1, a representative of the US_NmUC clade strains (S1 Fig). Both SNPs were present in all of the US_NmUC isolates. We also evaluated if 184 invasive NM strains, 9 urethritis/proctitis-associated NM strains from Europe [21, 22, 36], and 913 NG strains available in PubMLST encoded *norB* alleles and, if so, contained the US_NmUC associated SNPs [27]. We identified *norB* alleles in 41 of the invasive NM strains, 7 of the European urethritis/proctitis- associated NM strains, and in all of the NG strains. The G431A and C1782T SNPs were not present in any of the invasive or European urethritis/proctitis-associated NM strains. Ten of the NG strains had the C1782T SNP, but none of the NG strains had the G431A SNP.

**Fig 1:**
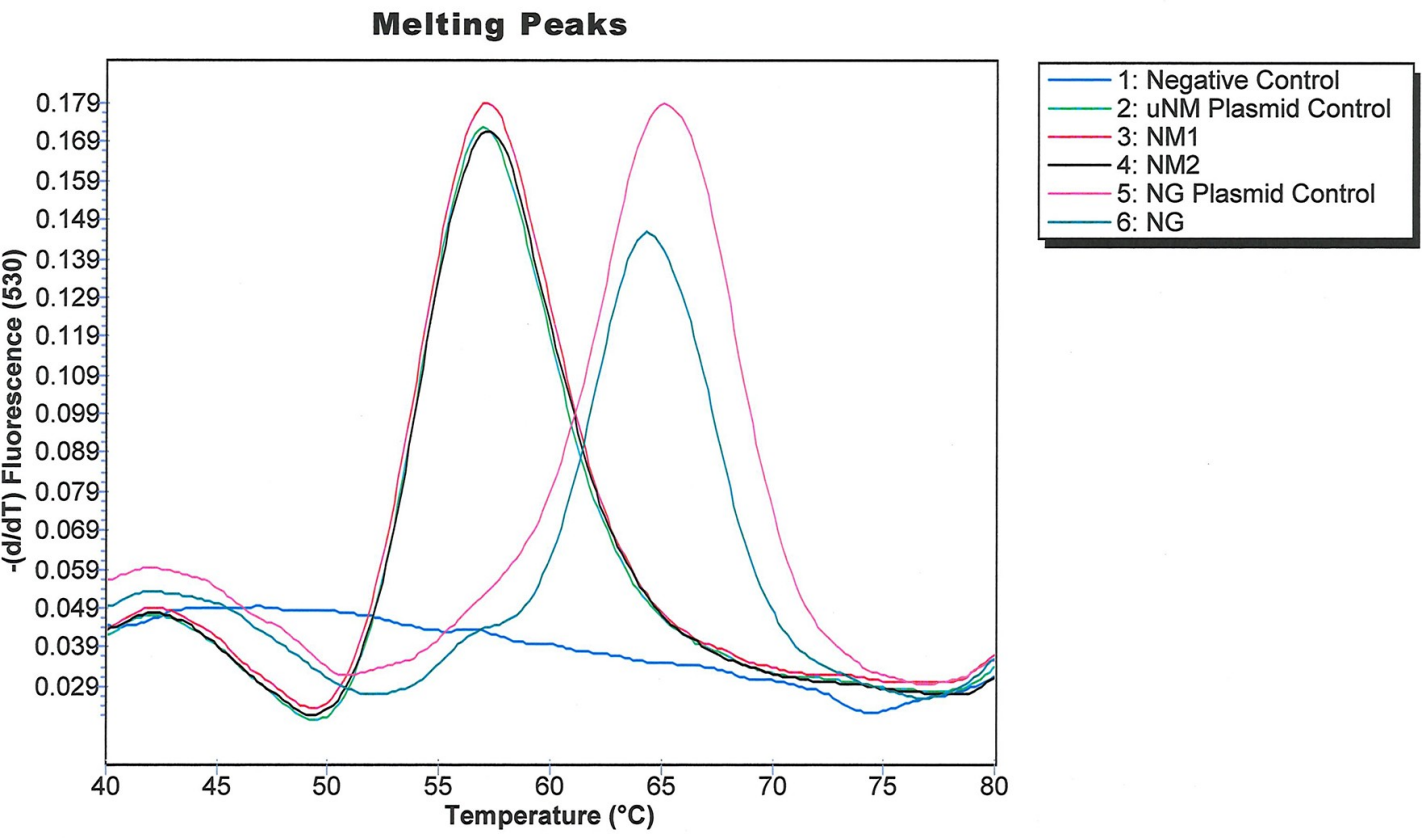
Melting curves of two local US_NmUC isolates (NM1 and NM2), the NG positive control, and another local NG isolate. The shift in melt curve peaks were clearly distinguishable, and separated by approximately 10°C.

The G431A SNP at position 431 causes a larger change in melting temperature than the C1782T SNP. Since our comparative genomic analysis also indicated that the G431A SNP perfectly differentiated the US_NmUC clades strains from invasive NM, European NM urethritis/proctitis, and NG strains, we designed a Simpleprobe® assay to target G431A using Applied Science Light Cycler Probe design software [31]. We performed a melting curve analysis to assess if the Simpleprobe® assay could differentiate the US_NmUC and NG *norB* alleles (Fig 1). NG *norB* melted between 65°C and 67°C, whereas NM1 *norB* melted between 55°C and 57°C.

We assessed the limit of detection (LOD) of our assay by spiking mock urine and vaginal swab matrices with serial dilutions of plasmids pNG-*norB* and pNM1-*norB*. Final plasmid concentrations ranged from 25 to 1000 copies/ml in the urine matrices, and 250–4000 copies/ml in the vaginal swab matrices. We extracted DNA from the contrived specimens using the Roche Cobas 4800 automated extraction platform and performed the SimpleProbe® assay on the LightCycler 2.0 and z480. We observed melting peaks at the predicted temperatures, confirming that the SimpleProbe® assay could discriminate NG and US_NmUC *norB* alleles in relevant matrices and on both PCR platforms. The limit of detection (LOD) was matrix and platform dependent. The lower LOD on the Light Cycler 2.0 platform was 50 copies/ml with urine and 500 copies/ml with vaginal swabs. On the z480 platform, the lower LOD was 100 copies/ml with urine and 1000 copies/ml with vaginal swabs.

### Cross-reaction with other urogenital microorganisms

**Fig 2:**
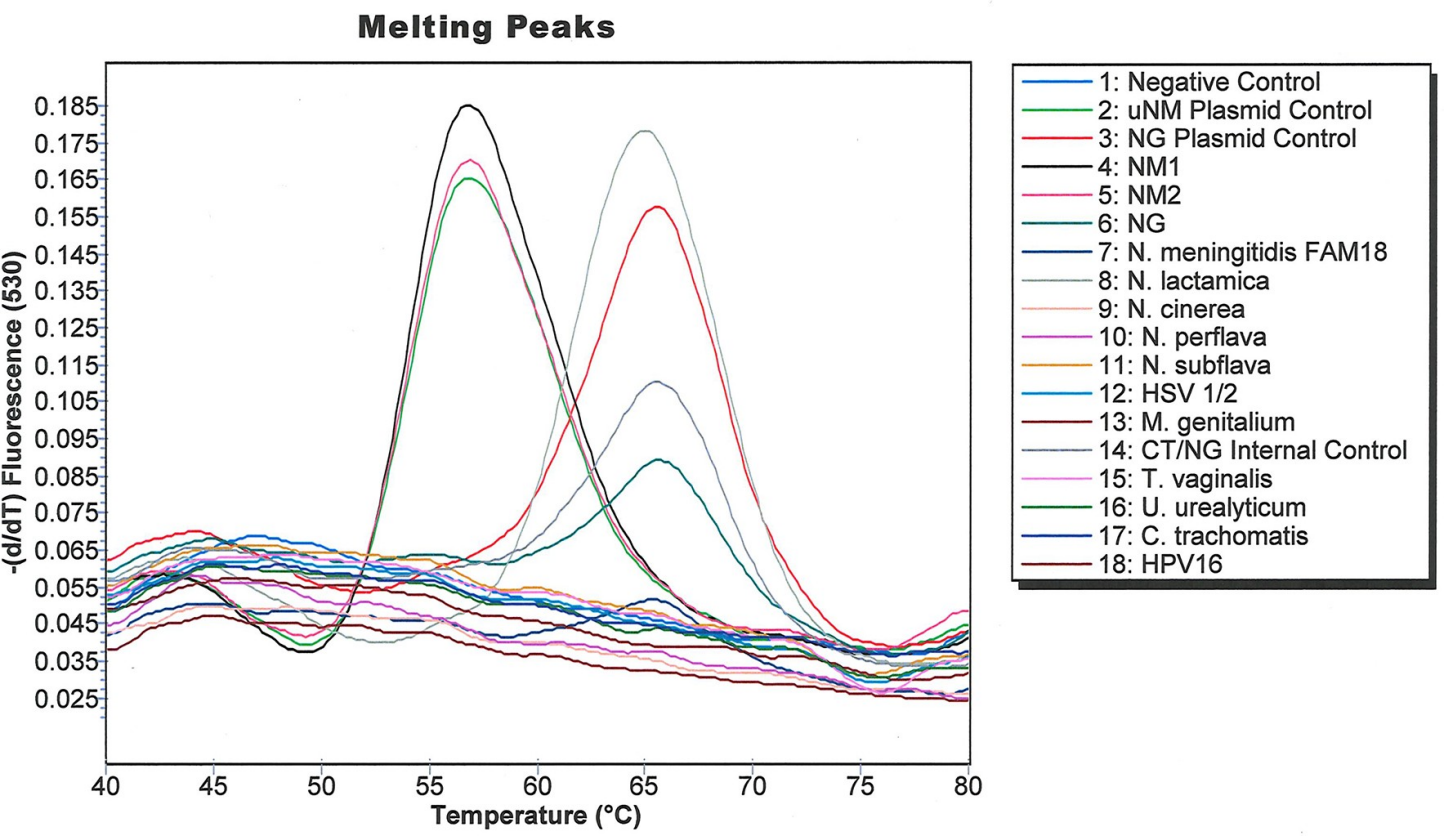
Amplification curves generated when the SimpleProbe® US_NmUC assay was tested against various microorganisms that can colonize the urogenital tract.

A diverse array of commensal [37, 38] as well as pathogenic microorganisms can sometimes be found in the distal male urethra, so we tested if high loads of DNA from some common urogenital microorganisms interfered with assay performance. Extracted DNA from various organisms including *C*. *trachomatis*, *T*. *vaginalis*, *M*. *genitalium*, *N*. *meningitidis*, *N*. *cinerea*, *N*. *lactamica*, *N*. *perflava*, *N*. *subflava*; genomic DNA from *U*. *urealyticum*, HSV 1, HSV2, HPV 16; or the control plasmids pNM1-*norB* and pNG-*norB* was tested in the Simpleprobe® assay. Contrived specimens spiked with the two US_NmUC isolates (NM1 and NM2), and the uNM plasmid control plasmid yielded a melting peak around 57°C, whereas none of the contrived specimens spiked with other *Neisseria* species or other urogenital microorganisms yielded a melting peak near this temperature (Fig 2). Contrived specimens spiked with NG, *N*. *lactamica* and the controls containing NG, or the NG plasmid yielded a melting peak around 66°C, whereas none of the other microorganisms amplified. Taken together, the clearly separated melting peaks showed complete predictive concordance with NG and US_NmUC strain calls.

### The US_NmUC Simpleprobe® assay is insensitive to high concentrations of NG *norB*

**Fig 3:**
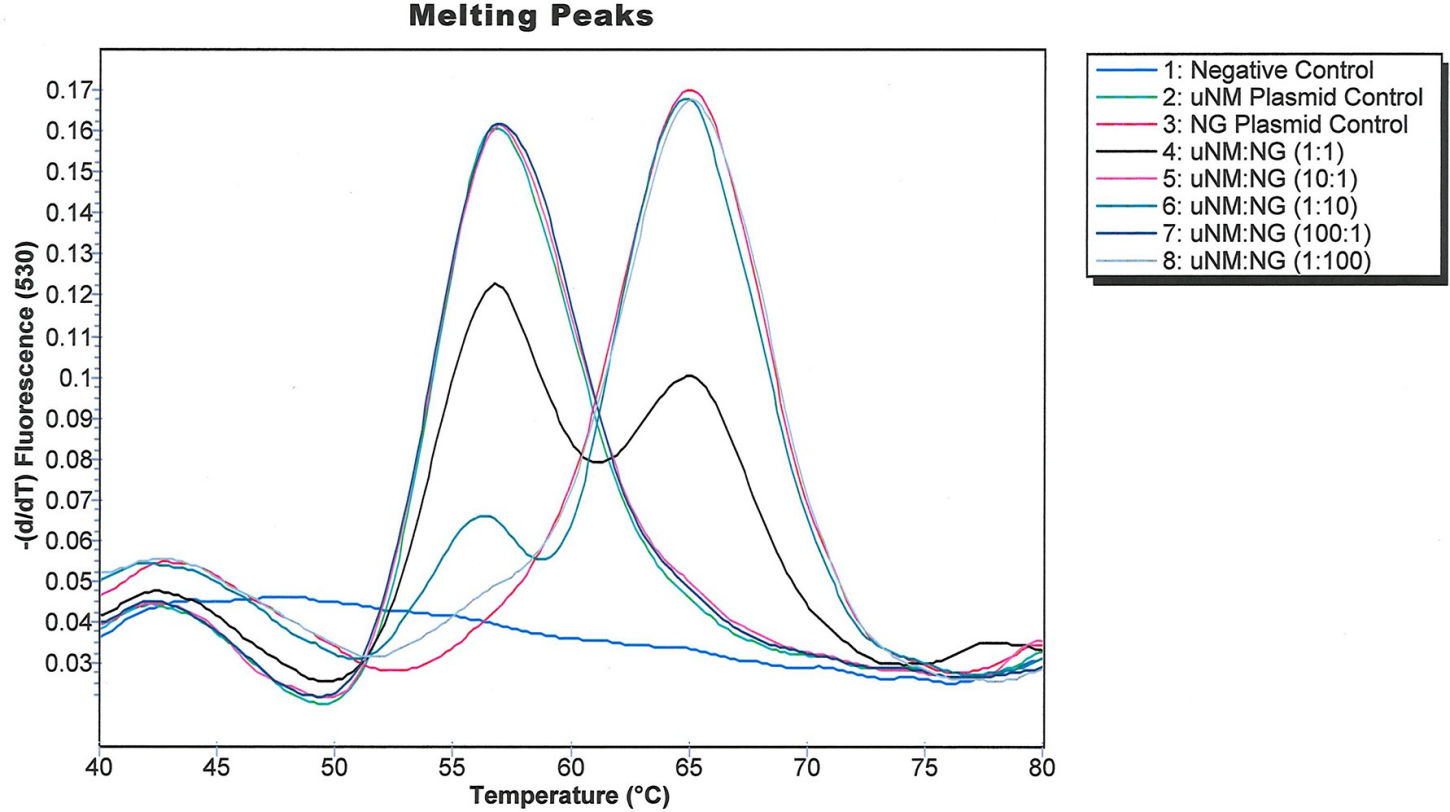
Amplification curves generated when the SimpleProbe® US_NmUC assay was tested against various dilutions of NG and NM.

The assay had a low LOD and yielded distinct melting peaks for NM1 and NG when these organisms were present singly in relevant matrices (Fig 1). Since concurrent infection with multiple STIs is common in men with urethritis, we tested the effects of different ratios of pNG-*norB* and pNM1-*norB* on the assay performance (Fig 3). The amplitudes of the NM1 and NG amplification peaks reflected the ratios of the corresponding templates in the reaction mixtures, and the US_NmUC and NG *norB* melting peaks were distinct at ratios ranging from 1:100 to 100:1 of pNG-*norB* and pNM1-*norB*. This result confirmed that the US_NmUC Simpleprobe® assay can differentiate US_NmUC in NG co-infected specimens.

### Validation of the SimpleProbe® US_NmUC assay using clinical specimens

**Fig 4:**
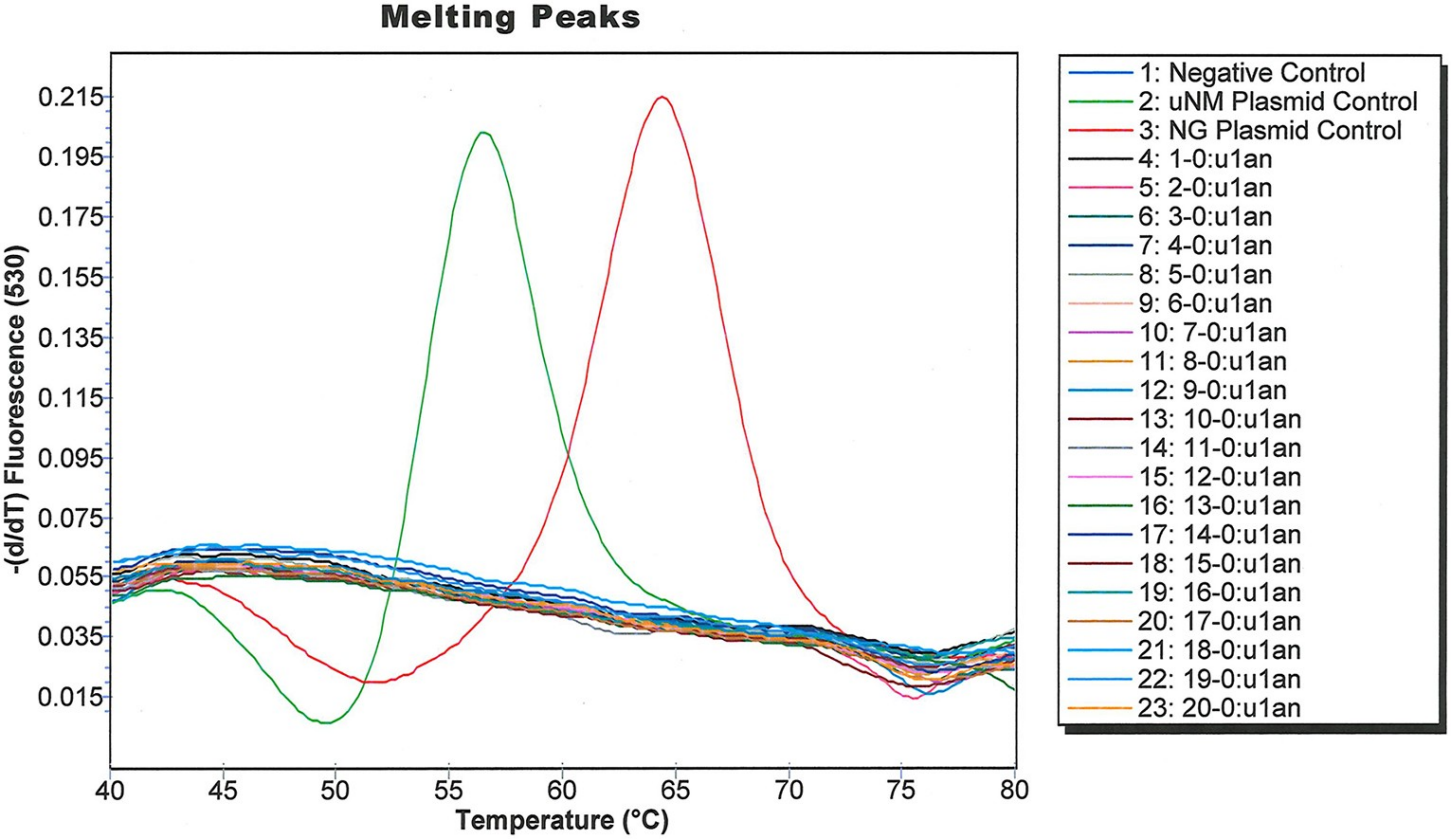
Amplification curves generated from specimens of the first 20 men in the IUMP cohort using the SimpleProbe® US_NmUC assay.

**Fig 5:**
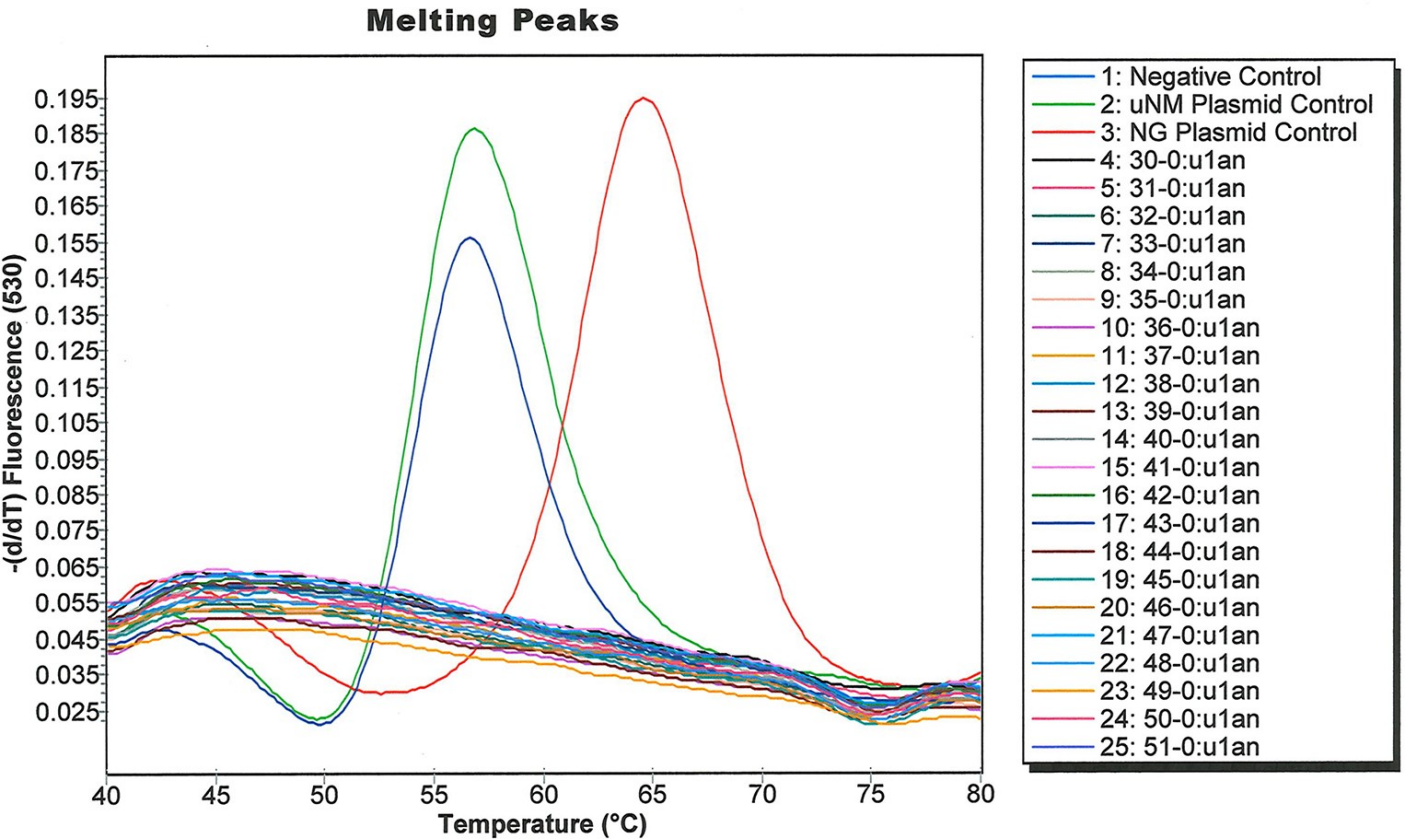
Amplification curve of one IUMP participant who tested positive for uNM in a separate group of 20 men.

**Fig 6:**
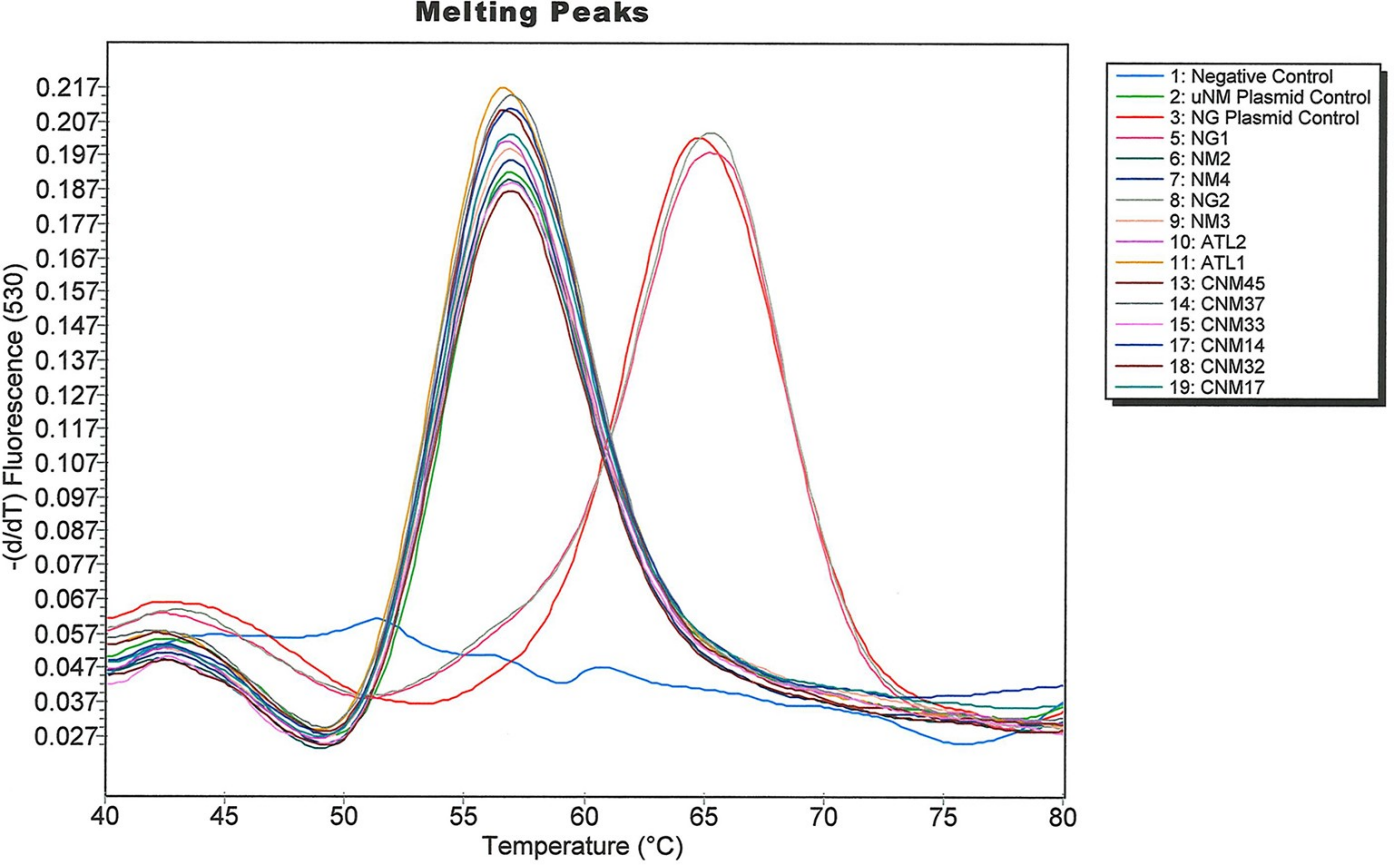
Amplification curves generated using a panel of US_NmUC isolates from geographically diverse case-clusters.

We used archived clinical specimens from the IUMP study [12, 33] to validate our assay. Urines were available from 241 men (enrolled between 08-04-2016 to 07-13-2018), including 127 NGU cases and 114 healthy controls which tested negative for NG. Deep shotgun metagenomic sequencing of urethral swab specimens from the same men, to an average depth of 7 gigabases, identified one case (Case 43) whose specimen contained a high proportion of US_NmUC sequences. Analysis of the same specimen collection using a Taqman PCR targeting Neisserial *metA* [35] identified three positive specimens, case 43, case 110, and healthy control 1062. PCR amplification and sequencing of *norB* from the swabs and urines from case 43, case 110, and control 1062 confirmed that case 43 was infected with US_NmUC, whereas case 110 and control 1062 were colonized with commensal NM strains. When the entire specimen collection was analyzed with the US_NmUC assay by a blinded technician, case 43 also yielded the expected amplification curve for US_NmUC, and the NG plasmid control yielded the expected melting peak for NG; whereas all of the other specimens yielded no amplification. To ensure that the lack of *metA* positive samples was not due to a lack of assay sensitivity, we performed the SimpleProbe® assay on the first 20 IUMP samples (Fig 4) and on a subsequent 22 samples that included one sample that was positive for *metA* (Fig 5). None of the *metA* negative samples tested positive in the SimpleProbe® assay. We also validated the assay using clinical isolates collected from various sites, including eleven whole genome sequenced US_NmUC isolates from Indianapolis, IN [25], Atlanta, GA [26], and Columbus, OH [24]. All of the US_NmUC isolates tested positive in the *metA* assay and yielded the expected amplification curves in the SimpleProbe® assay (Fig 6).

## Conclusions

We developed a simple and sensitive assay for US_NmUC clade strains that does not require microbial culture or genome sequencing. Assay performance was robust with US_NmUC clade strains isolated from a variety of clinical sites, male clinical urine and urethral specimens, and contrived vaginal specimens. The assay could easily be adapted for use with other types of clinical specimens and be compatible with the high throughput testing platforms present in contemporary diagnostic laboratories. This assay would facilitate future epidemiological studies to determine if US_NmUC clade strains can be carried asymptomatically, mechanisms of strain transmission, and their pathogenic potential. Our study has multiple limitations. First, we did not validate the assay for extragenital specimens or female clinical specimens, and were only able to include one unprocessed clinical specimen in our analysis. Optimization of the assay for use with extragenital specimens is an important future direction because there is evidence that the US_NmUC strains are transmitted via oral sexual exposures [39, 40]. Related to this issue, since there have been no prospective studies of US_NmUC prevalence, few properly consented and unprocessed specimens are currently available for US_NmUC testing. We hope that development of a rapid and simple test will inspire testing of retrospectively banked specimens and facilitate prospective studies of the US_NmUC clade strains. Finally, since our assay targets a single SNP, and it is unclear if US_NmUC mutants that had other bases at this site would be less fit, our assay would also miss US_NmUC that acquire mutations at other sites. Thus, a future direction is to identify additional similarly discriminating targets that could be included in a multi-plex assay format.

## Supporting information

S1 Fig *norB* DNA sequence alignment by clustalW. Two *N*. *gonorrheae* (NG) strains (FA1090, NCCP11945), one urethrotropic NM isolate (NM1), one Nm urethral isolate, and one *N*. *lactamica* (Nl) strain are included in the alignment. Sequences unique exclusively to the urethrotropic NM isolate are marked in bold red. The target SNP for our assay is boxed.
(DOCX)
