# Supplementary material for: Development of a SimpleProbe real-Time PCR Assay for rapid detection and identification of the US novel urethrotropic clade of Neisseria meningitidis ST-11 (US_NmUC)
Source: PLoS One. 2020 Feb 10;15(2):e0228467. doi: 10.1371/journal.pone.0228467 (PMC7010270; doi:10.1371/journal.pone.0228467)
Supplement: S1 Fig — Two N. gonorrheae (NG) strains (FA1090, NCCP11945), one urethrotropic NM isolate (NM1), one Nm urethral isolate, and one N. lactamica (Nl) strain are included in the alignment. Sequences unique exclusively to the urethrotropic NM isolate are marked in bold red. The target SNP for our assay is boxed. (DOCX) [file pone.0228467.s001.docx]

**Supplemental Figure**

**Fig S1**. *norB* DNA sequence alignment by clustalW.

CLUSTAL W (1.83) multiple sequence alignment

NG_NCCP11945 TTATTTGTCGCGGCCGAATACGATTTTAGTGGCTTGGATGGCAACGCAGATTGCACCGCCGATAAAGATTAAGTCGGGGG

NG_FA1090 TTATTTGTCGCGGCCGAATACGATTTTAGTGGCTTGGATGGCAACGCAGATTGCACCGCCGATAAAGATTAAGTCGGGGG

uNM_NM1 TTATTTGTCGCGGCCGAATACGATTTTAGTGGCTTGGATGGCAACGCAGATTGCACCGCCGATAAAGATTAAGTCGGGGG

NM_LNP26948 TTATTTGTCGCGGCCGAATACGATTTTAGTGGCTTGGATGGCAACGCAGATTGCACCGCCGATAAAGACCAAGTCAGCTG

NM_FAM18 TTATTTGTCGCGGCCGAATACGATTTTAGTGGCTTGGATGGCAACGCAGATTGCACCGCCGATAAAGACCAAGTCAGCTG

Nlac_020-06 TTATTTGTCCCGGCCGAATACGATTTTAGTGGCTTGGATGGCAACGCAGATTGCACCGCCGATAAAGATCAAGTCGGCGG

********* ********************************************************** ***** * *

NG_NCCP11945 CAGTGCGTACCCAGCGCAGGGTGTCGAGGATTTCCATTTGCAGGAATTCTTCGCTGCGGGCATACCACAAACCGTGCGTG

NG_FA1090 CAGTGCGTACCCAGCGCAGGGTGTCGAGGATTTCCATTTGCAGGAATTCTTCGCTGCGGGCATACCACAAACCGTGCGTG

uNM_NM1 CAGTGCGTACCCAGCGCAGGGTGTCGAGGATTTCCATTTGCAGGAATTCTTCGCTGCGGGCATACCACAAACCGTGCGTG

NM_LNP26948 CCGTACGTACCCAACGCAAGGTGTCGAGGATTTCCATTTGCAGGAACTCTTCGCTGCGTGCATACCACAGGCGGTGCGTG

NM_FAM18 CCGTACGTACCCAACGCAAGGTGTCGAGGATTTCCATTTGCAGGAACTCTTCGCTGCGTGCATACCACAGGCCGTGCGTG

Nlac_020-06 CAGTACGAACCCAGCGCAGGGTGTCGAGGATTTCCATTTGCAGGAACTCTTCGCTGCGGGCATACCACAGACCGTGCGTG

* ** ** ***** **** *************************** *********** ********** * *******

NG_NCCP11945 ATAGAGGCGTATGCCTGAATCACGCCGACAGGCAGCAGGCTGATGGCAATCATACCGACCAAGCCGCCGTTGAGCAGCCA

NG_FA1090 ATAGAGGCGTATGCCTGAATCACGCCGACAGGCAGCAGGCTGATGGCAATCATACCGACCAAGCCGCCGTTGAGCAGCCA

uNM_NM1 ATAGAGGCGTATGCCTGAATCACGCCGACAGGCAGCAGGCTGATGGCAATCATACCGACCAAGCCGCCGTTGAGCAGCCA

NM_LNP26948 ATGGAGGCGTATGCCTGAATCACGCCAACCGGCAACAGGCTGATGGCAATCATACCGACCAAGCCGCCGTTGAGCAGCCA

NM_FAM18 ATGGAGGCGTATGCCTGAATCACGCCAACCGGCAACAGGCTGATGGCAATCATACCGACCAAGCCGCCGTTGAGCAGCCA

Nlac_020-06 ATGGAGGCGTATGCCTGAATCGCGCCGACAGGCAGCAGGCTGATGGCAATCATACCGACCAAGCCGCCGTTGAGCAACCA

** ****************** **** ** **** ***************************************** ***

NG_NCCP11945 GAAGCCCCAAGTCATCAGTTTGTCGTCAAACCGCGCGTTCGGTTTCAAGTAGCGCGCAACCAACAATACGAAGCCCAATG

NG_FA1090 GAAGCCCCAAGTCATCAGTTTGTCGTCAAACCGCGCGTTCGGTTTCAAGTAGCGCGCAACCAACAATACGAAGCCCAATG

uNM_NM1 GAAGCCCCAAGTCATCAGTTTGTCGTCAAACCGCGCGTTCGGTTTCAAGTAGCGCGCAACCAACAATACGAAGCCCAATG

NM_LNP26948 GAAGCCCCAAGTCATCAGTTTGTCGTCAAACTGCGCGTTCGGTTTCAAATAACGGGCAACCAGCAATACGAAGCCCAATG

NM_FAM18 GAAGCCCCAAGTCATCAGTTTGTCGTCAAACTGCGCGTTCGGTTTCAAATAACGGGCAACCAGCAATACGAAGCCCAATG

Nlac_020-06 GAAGCCCCAAGTCATCAGTTTGTCGTCAAACTGCGCGTTCGGTTTCAAGTAGCGTGCAACCAGCAATACGAAGCCCAATG

******************************* **************** ** ** ******* *****************

NG_NCCP11945 CCAAGAAACCGTACACACCGAACAAGGCGGCGTGCGCGTGAACGGCGGAAGTGTTCAAACCTTGGATATAGAACAGGGAA

NG_FA1090 CCAAGAAACCGTACACACCGAACAAGGCGGCGTGCGCGTGAACGGCGGAAGTGTTCAAACCTTGGATATAGAACAGGGAA

uNM_NM1 CCAAGAAACCGTACACACCGAACAAGGCGGCGTGCGCGTGAACGGCGGAAGTGTTCAAACCTTGGATATAGAACAGGGAA

NM_LNP26948 CCAAGAAACCGTACACACCGAACAAGGCGGCGTGCGCATGAACAGCAGAAGTATTCAAACCTTGGATATAGAACAGGGAA

NM_FAM18 CCAAGAAACCGTACACACCGAACAAGGCGGCGTGCGCATGAACAGCAGAAGTATTCAAACCTTGGATATAGAACAGGGAA

Nlac_020-06 CCAAGAAACCGTACACACCGAACAAGGCGGCGTGCGCGTGAACGGCAGAAGTGTTCAAACCTTGGATATAGAACAGGGAA

************************************* ***** ** ***** ***************************

NG_NCCP11945 ATCGGCGGATTGATCAGAAAGCCGAATACGCCGGCACCGATCATATTCCAAAAAGCGACTGCCACGAAGCACATCAGCGG

NG_FA1090 ATCGGCGGATTGATCAGAAAGCCGAATACGCCGGCACCGATCATATTCCAAAAAGCGACTGCCACGAAGCACATCAGCGG

uNM_NM1 ATCGGCGGATTGATCAGAAAGCCGAATACGCCGGCACCGATCATATTCCAAAAAGCGACTGCCACGAAGCACAT**T**AGCGG

NM_LNP26948 ATCGGCGGGTTAATCAGGAAACCGAATACACCGGCACCGATCATATTCCAAAAGGCGACTGCCACGAAGCACATCAGCGG

NM_FAM18 ATCGGCGGGTTAATCAGGAAACCGAATACACCGGCACCGATCATATTCCAAAAGGCGACTGCCACGAAGCACATCAGCGG

Nlac_020-06 ATCGGCGGATTGATCAGGAAGCCGAATACGCCGGCACCGATCATATTCCAAAAGGCGACTGCCACGAAGCACATCAGCGG

******** ** ***** ** ******** *********************** ******************** *****

NG_NCCP11945 CCAACGCAGGCGTTTCGCCCAGTCGGACAGGTGTTGGTAAGACCAGTGCTCGTATGCCTCGCGGCCCAGCAACACCAGCG

NG_FA1090 CCAACGCAGGCGTTTCGCCCAGTCGGACAGGTGTTGGTAAGACCAGTGCTCGTATGCCTCGCGGCCCAGCAACACCAGCG

uNM_NM1 CCAACGCAGGCGTTTCGCCCAGTCGGACAGGTGTTGGTAAGACCAGTGCTCGTATGCCTCGCGGCCCAGCAACACCAGCG

NM_LNP26948 CCAACGCAGGCGTTTCGCCCAGTCGGACAGGTGTTGGTAAGACCAATGCTCGTATGCTTCACGGCCCAGCAACACCAGCG

NM_FAM18 CCAACGCAGGCGTTTCGCCCAGTCGGACAGGTGTTGGTAAGACCAATGCTCGTATGCTTCACGGCCCAGCAACACCAGCG

Nlac_020-06 CCAACGCAGGCGTTTCGCCCAGTCGGACAGATGTTGGTAAGACCAATGCTCGTATGCTTCACGACCCAGCAACACCAGCG

****************************** ************** *********** ** ** ****************

NG_NCCP11945 GCACGACTTCCAAAGCGGAGAAGCAGGCGCCGATTGCCATAGAGGCGGAGGTAGAGCCGGAGAAGTACAGGTGGTGCAGC

NG_FA1090 GCACGACTTCCAAAGCGGAGAAGCAGGCGCCGATTGCCATAGAGGCGGAGGTAGAGCCGGAGAAGTACAGGTGGTGCAGC

uNM_NM1 GCACGACTTCCAAAGCGGAGAAGCAGGCGCCGATTGCCATAGAGGCGGAGGTAGAGCCGGAGAAGTACAGGTGGTGCAGC

NM_LNP26948 GCACGACTTCCAAAGCGGAGAAGCAGGCACCGATTGCCATAGAGGCGGAGGTAGAGCCGGAGAAGTACAGGTGGTGCAGC

NM_FAM18 GCACGACTTCCAAAGCGGAGAAGCAGGCACCGATTGCCATAGAGGCGGAGGTAGAGCCGGAGAAGTACAGGTGGTGCAGC

Nlac_020-06 GCACGACTTCCAAAGCGGAGAAGCAGGCGCCGATTGCCATAGAGGCGGAGGTAGAGCCGGAGAAGTACAGGTGGTGCAGC

**************************** ***************************************************

NG_NCCP11945 GTGCCCGGAACGCCGCCCAACATAAAGATGGCGGCAGCGGCCAAAGTGGAGGCAGTGGCGGTACTGCGGCGGACAAAGCC

NG_FA1090 GTGCCCGGAACGCCGCCCAACATAAAGATGGCGGCAGCGGCCAAAGTGGAGGCAGTGGCGGTACTGCGGCGGACAAAGCC

uNM_NM1 GTGCCCGGAACGCCGCCCAACATAAAGATGGCGGCAGCGGCCAAAGTGGAGGCAGTGGCGGTACTGCGGCGGACAAAGCC

NM_LNP26948 GTACCCGGAACGCCGCCCAACATAAAGATGGCGGCAGCGGCCAAAGTAGAAGCTGTGGCGGTACTGCGGCGGACAAAGCC

NM_FAM18 GTACCCGGAACGCCGCCCAACATAAAGATGGCGGCAGCGGCCAGAGTAGAAGCTGTGGCGGTACTGCGGCGGACAAAGCC

Nlac_020-06 GTACCGGGAACGCCGCCCAACATAAAGATGGCGGCAGCAGCCAAAGTGGAGGCAGTGGCGGTACTGCGGCGGACAAAGCC

** ** ******************************** **** *** ** ** **************************

NG_NCCP11945 CATATTGTAGAAGACAAAGGCAAAGGCGGCAGTGGCAAATACTTCAAAGAAGCCTTCCACCCACAGGTGGACCACCCACC

NG_FA1090 CATATTGTAGAAGACAAAGGCAAAGGCGGCAGTGGCAAATACTTCAAAGAAGCCTTCCACCCACAGGTGGACCACCCACC

uNM_NM1 CATATTGTAGAAGACAAAGGCAAAGGCGGCAGTGGCAAATACTTCAAAGAAGCCTTCCACCCACAGGTGGACCACCCACC

NM_LNP26948 CATATTGTAGAAGACAAATGCGAAGGCGGCAGTGGCAAATACTTCAAAGAAGCCTTCCACCCACAGGTGAACCACCCACC

NM_FAM18 CATATTGTAGAAGACAAAGGCAAAAGCGGCAGTGGCAAATACTTCAAAGAAGCCTTCCACCCACAGGTGAACCACCCACC

Nlac_020-06 CATATTGTAGAAGACAAAGGCAAAGGCGGCAGTGGCAAATACTTCAAAGAAGCCTTCCACCCACAGGTGAACCACCCACC

****************** ** ** ******************************************** **********

NG_NCCP11945 AACGCCAGTATTCCATTACGGCAATCGGGGATTTTTCGCCATAGAACAGGCCCGGTGCGTAGAACACGCCCACGCCGACC

NG_FA1090 AACGCCAGTATTCCATTACGGCAATCGGGGATTTTTCGCCATAGAACAGGCCCGGTGCGTAGAACACGCCCACGCCGACC

uNM_NM1 AACGCCAGTATTCCATTACGGCAATCGGGGATTTTTCGCCATAGAACAGGCCCGGTGCGTAGAACACGCCCACGCCGACC

NM_LNP26948 AACGCCAGTATTCCATTACGGCAATCGGGGATTTCTCGCCATAGAACAGTCCCGGTGCGTAGAATACGCCCACACCGACC

NM_FAM18 AACGCCAGTATTCCATTACGGCAATCGGGGATTTCTCGCCATAGAACAGTCCCGGTGCGTAGAATACGCCCACACCGACC

Nlac_020-06 AGCGCCAGTATTCCATTACGGCAATCGGGGATTTTTCGCCATAGAACAGTCCCGGTGCGTAGAACACGCCCACACCGACC

* ******************************** ************** ************** ******** ******

NG_NCCP11945 ATAGAAGCGACAAAGATTGCCAGCAGGTTTTTGTCCACGCCTTTTTCTTTGAAGGCGGAAACCGTGCAGCGCAACATCAG

NG_FA1090 ATAGAAGCGACAAAGATTGCCAGCAAGTTTTTGTCCACGCCTTTTTCTTTGAAGGCGGAAACCGTGCAGCGCAACATCAG

uNM_NM1 ATAGAAGCGACAAAGATTGCCAGCAAGTTTTTGTCCACGCCTTTTTCTTTGAAGGCGGAAACCGTGCAGCGCAACATCAG

NM_LNP26948 ATAGAAGCGACAAAGATTGCCAGCAGGTTTTTGTCCACGCCTTTTTCTTTGAAGGCGGAAACCGTGCAACGCAACATCAG

NM_FAM18 ATAGAAGCGACAAAGATTGCCAGCAGGTTTTTGTCCACGCCTTTTTCTTTGAAGGCGGAAACCGTGCAACGCAACATCAG

Nlac_020-06 ATAGAAGCGACAAAGATTGCCAGCAGGTTTTTATCCACGCCTTTTTCTTTGAAGGCGGAAACCGTGCAGCGCAACATCAG

************************* ****** *********************************** ***********

NG_NCCP11945 GAACAGCCACAACAGCAGTCCGACCATCAAAAGGAGTTGCCAGAAACGTCCCAAATCGAGGTATTCGTAACCTTGGTGTC

NG_FA1090 GAACAGCCACAACAGCAGTCCGACCATCAAAAGGAGTTGCCAGAAACGTCCCAAATCGAGGTATTCGTAACCTTGGTGTC

uNM_NM1 GAACAGCCACAACAGCAGTCCGACCATCAAAAGGAGTTGCCAGAAACGTCCCAAATCGAGGTATTCGTAACCTTGGTGTC

NM_LNP26948 GAACAGCCACAACAGCAGGCCGACCATCAGCAGGAGTTGCCAGAAACGTCCCAAATCGAGGTATTCGTAACCTTGGTGTC

NM_FAM18 GAACAGCCACAACAGCAGGCCGACCATCAGCAGGAGTTGCCAGAAACGTCCCAAATCGAGGTATTCGTAACCTTGGTGTC

Nlac_020-06 GAACAGCCACAACAGCAGGCCGACCATCAACAGGAGTTGCCAGAAACGTCCCAAATCGAGGTATTCGTAACCTTGGTGTC

****************** ********** *************************************************

NG_NCCP11945 CGAACCAGAAGTTAAATTCGGGGGGAAGGATGTGCGTCAACGCGAAGAAGTTGCCCGCGTAAGAACCGCCGACCACGATG

NG_FA1090 CGAACCAGAAGTTAAATTCGGGAGGAAGGATGTGCGTCAACGCGAAGAAGTTGCCCGCGTAAGAACCGCCGACCACGATG

uNM_NM1 CGAACCAGAAGTTAAATTCGGGAGGAAGGATGTGCGTCAACGCGAAGAAGTTGCCCGCGTAAGAACCGCCGACCACGATG

NM_LNP26948 CGAACCAGAAGTTAAATTCCGGGGGAAGGATGTGCGTCAACGCGAAGAAGTTGCCCGCGTAAGAACCGCCGACCACGATG

NM_FAM18 CGAACCAGAAGTTAAATTCCGGGGGAAGGATGTGCGTCAACGCGAAGAAGTTGCCCGCGTAAGAGCCGCCGACCACGATG

Nlac_020-06 CGAACCAGAAGTTAAATTCGGGGGGAAGGATGTGCGTCAACGCGAAGAAGTTGCCCGCGTAAGAACCGCCGACCACGATA

******************* ** ***************************************** **************

NG_NCCP11945 AAGAGGGCGATATAGAGGAAGTTCACGCCTGCACGTTGGAACTTGGGATCTTTGCCGCCGTTGACAATCGGCGCGAGGAA

NG_FA1090 AAGAGGGCGATATAGAGGAAGTTCACGCCTGCACGTTGGAACTTGGGATCTTTGCCGCCGTTGACAATCGGCGCGAGGAA

uNM_NM1 AAGAGGGCGATATAGAGGAAGTTCACGCCTGCACGTTGGAACTTGGGATCTTTGCCGCCGTTGACAATCGGCGCGAGGAA

NM_LNP26948 AAGAGGGCGATATAGAGGAAGTTTACGCCGGCACGTTGGAACTTGGGATCTTTACCGCCGTTGACAATCGGCGCGAGGAA

NM_FAM18 AAGAGGGCGATATAGAGGAAGTTCACGCCTGCACGTTGGAACTTGGGATCTTTGCCGCCGTTGACAATCGGCGCAAGGAA

Nlac_020-06 AACAGGGCGATATAGAGGAAGTTCACGCCTGCACGTTGGAACTTGGGATCTTTGCCGCCGTTGACAATCGGCGCGAGGAA

** ******************** ***** *********************** ******************** *****

NG_NCCP11945 CAAACCTGCCGTCAAAAAGCCGGTTGCAATCCAGAAGATGGCGGATTGGATGTGCCAAGTACGGGTCAGGGCGTAGGGGA

NG_FA1090 CAAACCTGCCGTCAAAAAGCCGGTTGCAATCCAGAAGATGGCGGATTGGATGTGCCAAGTACGGGTCAGGGCGTAGGGGA

uNM_NM1 CAAACCTGCCGTCAAAAAGCCGGTTGCAATCCAGAAGATGGCGGATTGGATGTGCCAAGTACGGGTCAGGGCGTAGGGGA

NM_LNP26948 CAAACCTGCCGTCAAAAAGCCGGTTGCAATCCAGAAGATGGCTGATTGGATGTGCCAAGTACGGGTCAGGGCATAAGGGA

NM_FAM18 CAAACCTGCCGTCAAAAAGCCGGTTGCAATCCAGAAGATGGCGGATTGGATGTGCCAAGTACGGGTCAGGGCGTAAGGGA

Nlac_020-06 CAAACCTGCCGTCAAAAAGCCGGTTGCAATCCAGAAGATGGCGGATTGGATGTGCCAAGTACGGGTCAGGGCATAGGGGA

****************************************** ***************************** ** ****

NG_NCCP11945 ACCAGTCGGACATTTCAAAGCCCAACGCCTCGTCAATGCCGTAGAAACCCTGACCTTCGACGGTGTAGTGCGCGGTCAGG

NG_FA1090 ACCAGTCGGACATTTCAAAGCCCAACGCCTCGTCAATGCCGTAGAAACCCTGACCTTCGACGGTGTAGTGCGCGGTCAGG

uNM_NM1 ACCAGTCGGACATTTCAAAGCCCAACGCCTCGTCAATGCCGTAGAAACCCTGACCTTCGACGGTGTAGTGCGCGGTCAGG

NM_LNP26948 ACCAGTCGGACATTTCAAAGCCCAACGCTTCGTCGATGCCGTAGAAACCCTGGCCTTCGACGGTGTAGTGCGCGGTCAGT

NM_FAM18 ACCAGTCGGACATTTCAAAGCCCAACGCCTCGTCAATGCCGTAGAAACCCTGACCTTCGACGGTGTAGTGCGCGGTCAGA

Nlac_020-06 ACCAGTCGGACATTTCAAAGCCCAACGCCTCATCGATGCCGTAGAAACCCTGACCTTCGACGGTGTAGTGCGCGGTCAGA

**************************** ** ** ***************** **************************

NG_NCCP11945 CCGCCCAGCAATACTTGTACCACAAACAGGGCGACCGTCAGGAAGACGTATTTGCCCAATGCTTTTTGCGAAGGGGTCAG

NG_FA1090 CCGCCCAGCAATACTTGTACCACAAACAGGGCGACCGTCAGGAAGACGTATTTGCCCAATGCTTTTTGCGAAGGGGTCAG

uNM_NM1 CCGCCCAGCAATACTTGTACCACAAACAGGGCGACCGTCAGGAAGACGTATTTGCCCAATGCTTTTTGCGAAGGGGTCAG

NM_LNP26948 CCGCCCAGCAATACTTGTACCACAAACAGGGCGACCGTCAGGAAGACGTATTTGCCCAATGCTTTTTGCGAAGGGGTCAG

NM_FAM18 CCGCCCAGCAATACTTGTACCACAAACAGGGCGACCGTCAGGAAGACGTATTTGCCCAATGCTTTTTGCGAAGGGGTCAG

Nlac_020-06 CCGCCCAGCAATACTTGTACCACAAACAGGGCGACCGTCAGGAAGACGTATTTGCCCAATGCTTTTTGCGAAGGGGTCAG

********************************************************************************

NG_NCCP11945 TTGGATTTTGGAAATCGGGTCTTCAGACGGCACTTCCACTTCCTCGTGTTTGGTCAGGAAGGAATAACCCCACATCAACA

NG_FA1090 TTGGATTTTGGAAATCGGGTCTTCAGACGGCACTTCCACTTCCTCGTGTTTGGTCAGGAAGGAATAACCCCACATCAACA

uNM_NM1 TTGGATTTTGGAAATCGGGTCTTCAGACGGCACTTCCACTTCCTCGTGTTTGGTCAGGAAGGAATAACCCCACATCAACA

NM_LNP26948 TTGGATTTTGGAAATCGGGTCTTCAGACGGCACTTCCACTTCCTCGTGTTTGGTCAAGAAGGAATAACCCCACATCAGCA

NM_FAM18 TTGGATTTTGGAAATCGGGTCTTCAGACGGCACTTCCACTTCCTCGTGTTTGGTCAGGAAGGAATAACCCCACATCAACA

Nlac_020-06 TTGGATTTTGGAAATCGGATCTTCAGCCGGGATTTCCACTTCCTCGTGTTTGGTCAGGAAGGAATAACCCCACATCAGCA

****************** ******* *** * *********************** ******************** **

NG_NCCP11945 AACCGATGCCCATCAAGAGCAGTACGACACTGGTAAACGACCACATGTAGTTTTCAGTAGTCGGTACATTGTTGATCAAA

NG_FA1090 AACCGATGCCCATCAAGAGCAGTACGACACTGGTAAACGACCACATGTAGTTTTCAGTAGTCGGTACATTGTTGATCAAA

uNM_NM1 AACCGATGCCCATCAAGAGCAGTACGACACTGGTAAACGACCACATGTAGTTTTCAGTAGTCGGTACATTGTTGATCAAA

NM_LNP26948 AACCGATGCCCATCAGCAGAAGAACAACGCTGGTGAATGACCACATATAGTTTTCAGTGGTCGGTACGTTGTTGATCAAA

NM_FAM18 AACCGATGCCCATCAGCAGAAGAACAACGCTGGTGAATGACCACATATAGTTTTCAGTGGTCGGTACGTTGTTGATCAAA

Nlac_020-06 AACCGATGCCCATCAGCAGAAGAACAACGCTGGTAAACGACCACATATAGTTTTCAGTGGTCGGCACATTGTTGATCAAA

*************** ** ** ** ** ***** ** ******** *********** ***** ** ************

NG_NCCP11945 GGCTCGTGCGGCCAGTTGTTGGTGTAGGTAAAGACCTCGCCGGGACGGTTGGTCGAAGCAGACCAAGAAGTCCAGAAGAA

NG_FA1090 GGCTCGTGCGGCCAGTTGTTGGTGTAGGTAAAGACCTCGCCGGGACGGTTGGTCGAAGCAGACCAAGAAGTCCAGAAGAA

uNM_NM1 GGCTCGTGCGGCCAGTTGTTGGTGTAGGTAAAGACCTCGCCGGGACGGTTGGTCGAAGCAGACCAAGAAGTCCAGAAGAA

NM_LNP26948 GGCTCGTGCGGCCAGTTGTTGGTATAAGTGAACGTCTCGTCAGGACGGTTGGTCGAAGCAGACCAAGAAGTCCAGAAGAA

NM_FAM18 GGCTCGTGCGGCCAGTTGTTGGTATAAGTGAACGTCTCGTCAGGACGGTTGGTCGAAGCAGACCAAGAAGTCCAGAAGAA

Nlac_020-06 GGCTCGTGCGGCCAGTTGTTGGTGTAGGTAAAGACCTCGCCGGGACGGTTGGTCGAAGCAGACCAAGAAGTCCAAAAGAA

*********************** ** ** ** **** * ******************************** *****

NG_NCCP11945 GAAGTCGAACAGTTTTTCACGCGCTTCTTGGCTTGGCAATGTGTTGTTTTTCATTGCAAAGTGTTCGCGGGTGGTTTGCA

NG_FA1090 GAAGTCGAACAGTTTTTCACGCGCTTCTTGGCTTGGCAATGTGTTGTTTTTCATTGCAAAGTGTTCGCGGGTGGTTTGCA

uNM_NM1 GAAGTCGAACAGTTTTTCACGCGCTTCTTGGCTTGGCAATGTGTTGTTTTTCATTGCAAAGTGTTCGCGGGTGGTTTGCA

NM_LNP26948 GAAGTCGAACAGTTTTTCACGCGCTTCTTGGCTTGGCAATGTGTTGTTTTTCATTGCAAAGTGTTCGCGGGTGGTTTGCA

NM_FAM18 GAAGTCGAACAGTTTTTCACGCGCTTCTTGGCTTGGCAATGTGTTGTTTTTCATTGCAAAGTGTTCGCGGGTGGTTTGCA

Nlac_020-06 GAAGTCGAACAGTTTTTCACGCGCTTCTTGGCTTGGCAATGTGTTGTTTTTCATTGCAAAGTGTTCGCGGGTGGTTTGCA

********************************************************************************

NG_NCCP11945 ACTTGGGATCATCGCCGTAAACGCCGTGATAGTAAGGCAGGATGCTTTCGATGGCTTTCACGCGCGTATCGCTGATGACG

NG_FA1090 ACTTGGGATCATCGCCGTAAACGCCGTGATAGTAAGGCAGGATGCTTTCGATGGCTTTCACGCGCGTATCGCTGATGACG

uNM_NM1 ACTTGGGATCATCGCCGTAAACGCCGTGATAGTAAGGCAGGATGCTTTCGATGGCTTTCACGCGC**A**TATCGCTGATGACG

NM_LNP26948 GCGCGGGATCATCGCCGTAAACACCGTGGTAATAAGGCAGGATGCTTTCGATGGCTTTCACGCGCGTATCGCTGATGACG

NM_FAM18 GCGCGGGATCATCGCCGTAAACACCGTGGTAATAAGGCAGGATGCTTTCGATGGCTTTCACGCGCGTATCGCTGATGACG

Nlac_020-06 GCTTGGGATCGTCGCCGTAAACGCCGTGGTAATAAGGCAGGATGCTTTCGATGGCTTTCACGCGCGTATCGCTGATGACG

* ****** *********** ***** ** ********************************* **************

NG_NCCP11945 ACGCTGCCGTCTTCTTTAATACGGCTTTGATTGCGGTATTCATCGGCCAAGCGGGTTTTCAGAACGGCTTGTTCTTCAGG

NG_FA1090 ACGCTGCCGTCTTCTTTAATACGGCTTTGATTGCGGTATTCATCGGCCAAGCGGGTTTTCAGAACGGCTTGTTCTTCAGG

uNM_NM1 ACGCTGCCGTCTTCTTTAATACGGCTTTGATTGCGGTATTCATCGGCCAAGCGGGTTTTCAGAACGGCTTGTTCTTCAGG

NM_LNP26948 ACGCTGCCGTCTTCTTTAATACGGCTTTGGTTGCGGTATTCGTCAGCCAGTCGGGTTTTCAGAACGGCTTGTTCTTCAGG

NM_FAM18 ACGCTGCCGTCTTCTTTAATACGGCTTTGGTTGCGGTATTCGTCAGCCAGTCGGGTTTTCAGAACGGCTTGTTCTTCAGG

Nlac_020-06 ACGCTGCCGTCTTCTTTCACACGGCTTTGGTTGCGGTATTCGTCAGCCAGGCGGGTTTTCAGAACGGCTTGTTCTTCAGG

***************** * ********* *********** ** **** ****************************

NG_NCCP11945 GGAAACTTCATCGAATTTTTTGCCGTAAGCCTGTTGCGCGGTCAAATCCAACCAGGCGGACAACTCACGATGCAGCCAGT

NG_FA1090 GGAAACTTCATCGAATTTTTTGCCGTAAGTCTGTTGCGCGGTCAAATCCAACCAGGCGGACAACTCACGATGCAGCCAGT

uNM_NM1 GGAAACTTCATCGAATTTTTTGCCGTAAGCCTGTTGCGCGGTCAAATCCAACCAGGCGGACAACTCACGATGCAGCCAGT

NM_LNP26948 GGAAACTTCATCGAATTTTTTGCCGTAAGTCTGTTGCGCGGTCAAATCCAACCAAGCGGACAACTCACGATGCAGCCAGT

NM_FAM18 GGAAACTTCATCGAATTTTTTGCCGTAAGTCTGTTGCGCGGTCAAATCCAACTATGCAACCAACTCACGATGCAGCCAGT

Nlac_020-06 GGAAACTTCATCAAATTTTTTGCCGTAAGTCTGTTGCGCGGTCAAATCCAACCAGGCAGACAACTCACGATGCAGCCAGT

************ **************** ********************** * ** ********************

NG_NCCP11945 CGGCCGTCCAATCCGGAGCCTGATATGCGCCGTGTCCCAGAATCGAACCGACTTCCATGCCGCCGGTAGTCTGCCACGCA

NG_FA1090 CGGCCGTCCAGTCCGGAGCCTGATATGCGCCGTGACCCAGAATCGAACCGACTTCCATGCCGCCGGTACTCTGCCACGCA

uNM_NM1 CGGCCGTCCAATCCGGAGCCTGATATGCGCCGTGTCCCAGAATCGAACCGACTTCCATGCCGCCGGTAGTCTGCCACGCA

NM_LNP26948 CCGCCGTCCAGTCCGGAGCCTGATATGCGCCGTGACCCAGAATCGAACCGACTTCCATACCGCCGGTACTCTGCCACGCA

NM_FAM18 CGGCCGTCCAGTCCGGAGCCTGATATGCGCCGTGACCCAGAATCGAACCGACTTCCATACCGCCGGTACTCTGCCACGCA

Nlac_020-06 CGGCCGTCCAGTCCGGAGCCTGATATGCGCCGTGACCCAAAATCGAACCGACTTCCATACCGCCGGTACTCTGCCATGCA

* ******** *********************** **** ****************** ********* ******* ***

NG_NCCP11945 GACTGACCTGCCAAAATATCGTCTTTCGTCATCAGCACTTTGCCTGATGCGGAAACGACCTGTTCGGGGTAAGGCGGGGC

NG_FA1090 GACTGACCTGCCAAAATATCGTCTTTCGTCATCAGCACTTTGCCGGATGCGGAAACGACCTGTTCGGGGTAAGGCGGGGC

uNM_NM1 GACTGACCTGCCAAAATATCGTCTTTCGTCATCAGCACTTTGCCGGATGCGGAAACGACCTGTTCGGGGTAAGGCGGGGC

NM_LNP26948 GACTGACCTGCCAAAATATCGTCTTTCGTCATCAGCACTTTGCCTGATGCGGAAACGACCTGTTCGGGGTAAGGCGGGGC

NM_FAM18 GACTGACCTGCCAAAATATCGTCTTTCGTCATCAGCACTTTGCCTGATGCGGAAACGACCTGTTCAGGGTAAGGCGGGGC

Nlac_020-06 GACTGACCTGCCAAAATATCGTCTTTCGTCATCAGCACTTTGCCTGATGCGGAAACGACCTGTTCGGGATAAGGCGGGGC

******************************************** ******************** ** ***********

NG_NCCP11945 TTTCTTATAAACCTCGCTGCCCATATAGCCAAGAATGGTAAAGCATACCGCCAGAACGGCAAACAGCAAGTACCACAGCT

NG_FA1090 TTTCTTATAAACCTCGCTGCCCATATAGCCAAGAATGGTAAAGCATACCGCCAGAACGGCAAACAGCAAGTACCACAGCT

uNM_NM1 TTTCTTATAAACCTCGCTGCCCATATAGCCAAGAATGGTAAAGCATACCGCCAGAACGGCAAACAGCAAGTACCACAGCT

NM_LNP26948 TTTCTTATAAACCTCGCTGCCCATATAGCCAAGAATGGTAAAGCATACCGCCAGAACGGCAAACAGCAAGTACCACAGCT

NM_FAM18 TTTTTTGTAAACCTCGCTGCCCATATAGCCAAGAATGGTAAAGCATACCGCCAGAACGGCAAACAGCAAGTACCATAGCT

Nlac_020-06 TTTCTTATAAACCTCGCTGCCCATATAGCCGAGAATGGTAAAGCATACCGCCAGAACGGCGAACAGCAAGTACCACAGCT

*** ** *********************** ***************************** ************** ****

NG_NCCP11945 TCTTGTACTGTCCCAT

NG_FA1090 TCTTGTACTGTCCCAT

uNM_NM1 TCTTGTACTGTCCCAT

NM_LNP26948 TCTTGTACTGTCCCAT

NM_FAM18 TCTTGTACTGTCCCAT

Nlac_020-06 TCTTATACTGTCCCAT

**** ***********
